# The Autonomic Regulation of Circulation and Adverse Events in Hypertensive Patients during Follow-Up Study

**DOI:** 10.1155/2019/8391924

**Published:** 2019-12-30

**Authors:** Oleg V. Mamontov, Andrey V. Kozlenok, Alexei A. Kamshilin, Evgeny V. Shlyakhto

**Affiliations:** ^1^Dept. of Circulation Physiology, Almazov National Medical Research Centre, St. Petersburg 197341, Russia; ^2^Dept. of Departmental Therapy, Pavlov First Saint Petersburg State Medical University, St. Petersburg 197022, Russia; ^3^Faculty of Applied Optics, ITMO University, St. Petersburg 197101, Russia

## Abstract

**Purpose:**

Comprehensive study of autonomic regulation assessed during follow-up could provide new detailed information about the risks stratification for hypertensive patients. Therefore, we investigated the associations of these indices with death, stroke, and revascularization during the follow-up observation of 55 patients.

**Methods:**

All patients were with target organ damage, and 27 of them had associated clinical conditions (ACC). Mean age of patients with and without ACC was 62.6 ± 4.2 and 51.9 ± 9.9 (mean ± SD) years, respectively. Follow-up was from 66 to 95 months. At entry, autonomic regulation was assessed by the tilt test, Valsalva maneuver, hand-grip test, and cold-stress vasoconstriction. Hemodynamic parameters were measured by continuous blood pressure monitoring, occlusion plethysmography, and electrocardiography. Re-examination of patients was carried out by questioning and physical and laboratory examination.

**Results:**

We found that fatal outcomes were associated with a lower Valsalva index (1.34 ± 0.16 vs. 1.69 ± 0.37, *P* < 0.05) and depressed cold vasoconstriction (0.20 ± 0.02 vs. 0.39 ± 0.16%, *P* < 0.05) and depressed cold vasoconstriction (0.20 ± 0.02 vs. 0.39 ± 0.16%, *P* < 0.05) and depressed cold vasoconstriction (0.20 ± 0.02 vs. 0.39 ± 0.16%, *P* < 0.05) and depressed cold vasoconstriction (0.20 ± 0.02 vs. 0.39 ± 0.16%, *P* < 0.05) and depressed cold vasoconstriction (0.20 ± 0.02 vs. 0.39 ± 0.16%, *P* < 0.05) and depressed cold vasoconstriction (0.20 ± 0.02 vs. 0.39 ± 0.16%, *P* < 0.05) and depressed cold vasoconstriction (0.20 ± 0.02 vs. 0.39 ± 0.16%,

**Conclusions:**

This study shows that such autonomic regulation indices as Valsalva index, blood pressure dynamics in the tilt test, cold-stress vasomotor reactivity, and BPV are important for prognosis of hypertension course.

## 1. Introduction

Hypertension is the most important factor worsening the prognosis and survival of patients with cardiovascular diseases [1]. However, it is known that not all hypertensive patients have equal chances for disease progression [2]. The factors of cardiometabolic risk are the most thoroughly investigated: a number of systemic publications are devoted to study of the prognosis of these factors for patients with cardiovascular diseases in developed countries [3]. At the same time, there are often studies in which autonomic regulation and parameters of hemodynamic were studied resulting in inclusion of respective indicators in the management for sudden death issued by the American Heart Association [4]. However, the prognosis of the patients was studied most often after the development of complications and concerned, as a rule, sudden death. In the vast majority of these studies, the markers that relate mainly to the regulation of the heart rhythm, such as the cardiac component of the arterial baroreflex, heart rate variability, heart rate turbulence, and alteration of the T-wave, were used [5]. Probably, these markers were selected due to commonly accepted study design in which the indices of the state of autonomous regulation were extracted from the data obtained in either the emergency or hospital.

The prognostic value of more specific markers of autonomic regulation, such as the reuptake of norepinephrine, using scintigraphy with iodine-123 meta-iodobenzylguanidine, has been studied only in a small number of papers [6, 7]. Basic information about significance of the autonomic nervous system for prognosis was obtained from the analysis of heart rate variability, concentration catecholamine measurement, and arterial baroreflex in patients with heart failure and myocardial infarction [8, 9]. However, vasomotor regulation of blood circulation has a major role in maintaining systemic hemodynamics, changes in which are also associated with a prognosis worsening. Munhoz et al. found that patients with chronic heart failure significantly more likely died during an 18-month follow-up if they had high muscle tone of the sympathetic nerve and low peripheral blood flow [10].

There are studies in which indicators relating to both cardiac and vasomotor neurogenic reactivity in patients with hypertension during follow-up were analyzed in the presence of diabetes [11] or with autonomic failure [12]. However, we have not found any work devoted to comprehensive study of prognostic value of the autonomic regulation in patients with essential hypertension but in absence of significant diabetic autonomic neuropathy and autonomic or heart failure. Nevertheless, prognosis of the increased mortality risk, the risk of developing cardiovascular complications associated with the progression of the hypertension, and a decrease in quality of patient life is very important.

The aim of this research was to evaluate the prognostic significance of the indices of autonomic regulation and hemodynamic parameters in hypertensive patients with target organ damage and with associated clinical conditions in the course of the follow-up observation.

## 2. Methods

### 2.1. Patients

This study enrolled 55 patients with essential hypertension. All patients underwent either inpatient or outpatient examinations at the Almazov National Medical Research Center, St. Petersburg. The criteria for inclusion of the patient in the study were his stable condition and regular therapy for at least 10 days. The exclusion criterion was the presence of a severe concomitant disease that was proven to affect the prognosis. The study plan was approved by the research ethical committee of the Almazov National Medical Research Center prior to the examinations. All patients provided their informed consent in the written form for participation in the study.

At the time of the initial examination, all subjects were classified as patients with target organ damage. About half of them had associated clinical conditions (ACCs). The average time of the follow-up observation was 7.1 ± 2.2 years (from 5.5 to 7.9 years). Average age of hypertensive patients with and without ACC was 62.6 ± 4.2 and 51.9 ± 9.9 years, respectively. Dominating associated clinical conditions for patients included in the study is given in Table 1. Most patients with associated conditions had coronary artery disease, including myocardial infarction and previous myocardial revascularization. Two patients had significant atherosclerosis of the brachycephalic arteries, and two patients had a stroke.

### 2.2. Autonomic Testing

During the initial examination, all patients underwent cardiac examination including echocardiogram recording and evaluation of cardiometabolic risk factors. Moreover, a comprehensive assessment of autonomic regulation of blood circulation was carried out for all patients according to the following sequence of tests:

1. Tilt test of a shortened protocol (10 min. at rest and 10 min. in orthostasis)
2. Valsalva maneuver
3. Hand-grip test
4. Cold stress-induced vasoconstriction for evaluation reaction of the forearm vessels while cooling the skin of the upper chest for 2 minutes

In addition, the spontaneous arterial baroreflex (ABR) and the spectral power of variability of both the heart rate and arterial blood pressure (BP) were estimated. Hemodynamic parameters were measured continuously by means of a BP monitor Finometer-Pro (Finapres Medical Systems, The Netherlands) with parallel electrocardiogram (ECG) recording. The forearm blood flow was measured by venous occlusion plethysmography using a Dohn air-filled cuff.

The secondary examination of the patients enrolled in the study was done by interviewing, physical examination, and reviewing medical records obtained on the basis of inpatient and outpatient analyses performed in the last three months before censoring. In the event of patient's death, a survey of his relatives was conducted. The questionnaire is shown in Table 2. The analysis of the relationship between the indices autonomous regulation at the time of the survey and outcomes based on the results of the questionnaire survey was carried out after interviewing and forming a database.

### 2.3. Statistical Analysis

To estimate the difference in the measured hemodynamic parameters or autonomic regulation indices between patient samples, we used the nonparametric Mann–Whitney test. Difference in qualitative and categorical signs was assessed by analyzing the contingency tables (Pearson criterion *χ*^2^). The level of *P* < 0.05  was considered as significant, whereas the level of 0.05 < *P* < 0.1 was considered as a tendency. The statistical analysis of the measured data was carried out by using the software Statistica 10 (StatSoft Russia).

## 3. Results

The study revealed that both the Valsalva index and diastolic blood pressure were significantly different in groups of patients with and without ACC. In addition, it was found that the parameter of cold vasoconstriction and heart rate are tended to differ as it is shown in Table 3.

In the course of questioning, it was found that five patients died during the follow-up (see Table 4). All deceased patients at the time of the initial examination had ACC, *χ*^2^ = 4.46; *P* < 0.05. In all cases, the primary cause of the death was cardiovascular disease progression. Only three patients had a new myocardial infarction: two of them initially had hypertension with ACC, whereas three patients had target organ damage only. New stroke was registered in six patients: four with ACC and two without. The revascularization of the coronary arteries has been performed to seven patients of whom six patients with ACC and one without, *χ*^2^ = 3.05; *P* < 0.08. At the time of enrollment, nine patients had concomitant type 2 diabetes mellitus. At the secondary examination, new cases of diabetes were identified in four patients, three of them with ACC, and one without.

Therefore, out of 27 hypertensive patients with target organ damage only, four patients have experienced ACC. Patients with ACC died and underwent revascularization more often, whereas there were no difference in the number of new cases of cardiovascular complications between patients with and without ACC.

### 3.1. Hemodynamics and Autonomic Regulation Associated with Fatal Outcome

Since all the dead during the observation period had ACC at the time of initial examination, the comparative analysis was performed only in the group of hypertensive patients with ACC. When assessing the relationship between baseline clinical data, autonomic regulation parameters, and hemodynamic parameters in groups with different outcomes, it was found that patients who had died by the time of the second examination/survey was elder than survivors: 77.4 ± 14.5 versus 61.3 ± 14.3 years; *P*=0.02.

Patients at the time of enrollment to the study had no signs of clinically significant heart failure, and only one patient had initial cardiomegaly, whereas the grade of chronic heart failure was estimated as I functional class (New York Heart Association). At the same time, the left ventricle ejection fraction was comparable in the group with fatal outcome (57.0 ± 5.4%) and in the group of survivors (59.6 ± 11.6%); *P* > 0.05. However, the total peripheral resistance (TPR) was observed (during the initial examination) to be significantly higher among the patients who had died: 1.36 ± 0.19 versus 0.86 ± 0.25 rel. un., *P* < 0.001. Moreover, there were a number of differences in the indices of autonomic regulation of blood circulation in deceased patients at the time of the initial examination. Patients of this group had a more pronounced decrease of diastolic BP in the orthostasis (−7.8 ± 8.0 mmHg) in contrast to the expected increase (1.5 ± 6.6 mmHg) in the group of survivors, *P*=0.011. Note that difference between the groups in the dynamics of diastolic BP was most clearly manifested in the initial period of the orthostasis (the first minute): −13.0 ± 2.5 versus −2.2 ± 8.6 mmHg, *P*=0.011. Moreover, variability of BP in the high-frequency (respiratory) range was much higher in the group of deceased patients as compared to another group: 18.2 ± 14.2 and 6.2 ± 4.2 mmHg^2^, respectively.

Contrary to expectations, deceased patients did not demonstrate a significant reduction in the magnitude of spontaneous ABR compared to the group of survivors: 4.4 ± 3.0 and 7.5 ± 6.5 ms/mmHg, respectively, *P* > 0.05. This was most likely due to insufficient sample size of the former group. However, the Valsalva index was smaller in the group of deceased patients: 1.34 ± 0.16 versus 1.69 ± 0.37 rel. un., *P* < 0.05. Moreover, weakening of cold vasoconstriction of the forearm vessels was observed in this group: 0.20 ± 0.02 versus 0.39 ± 0.16 rel. un., *P* < 0.05. The data indicate a high risk of death in the group with a decrease in cardiac and vasomotor efferent reactivity against a background of increased both peripheral vascular resistance at rest and BP variability in the respiratory range.

### 3.2. Hemodynamics and Autonomic Regulation Associated with New Stroke

It should be noted that none of the patients who had undergone stroke during the observation had this diagnosis at the time of the initial examination. Therefore, the analysis was performed only in a group of patients who had no stroke or hemodynamic significant atherosclerosis of brachiocephalic arteries previously. Four patients who died during the observation period did not have reliable data on the stroke. Therefore, they were excluded from the analysis. Consequently, 47 patients were analyzed. It was found that the age of stroke patients had a tendency to be higher than in the comparison group: 65.8 ± 13.8 versus 55.2 ± 13.2, *P*=0.07.

In addition, six patients who suffered stroke during the follow-up had specific reaction of BP on the orthostasis. The systolic BP increase in the orthostasis was observed in the stroke group, whereas it was the expected decrease of this parameter in the comparison group (41 patients): 8 ± 16 and −8 ± 12 mmHg, respectively, *P* < 0.01. It is worth noting that variability of the total systolic BP was higher in the group of patients suffered of stroke than in the comparison group (103 ± 51 and 65 ± 45 mmHg, *P* < 0.05), which was mainly due to very low-frequency spectral component (<0.04 Hz): 67 ± 40 and 37 ± 32 mmHg, respectively, *P* < 0.05. Therefore, the increased risk of stroke development is associated with aging, as well as with the hypertensive response on the orthostasis and high BPV.

### 3.3. Predictors of Hypertension Progression

As seen in Table 4, progression of the hypertension, which was defined as the appearance of associated clinical conditions in patients who did not have their previously, was noted in four cases.

By using comparative analysis, we have found that patients who transferred to the disease stage with ACC (four patients) were comparable by age with other patients in the group without ACC (twenty-three patients): 54.5 ± 9.3 versus 50.2 ± 13.7 years, respectively, *P*=0.55. In spite of comparable values of initial BP (130 ± 11 and 133 ± 15 mmHg, *P* > 0.05), patients with progressive of disease had significant increase of both diastolic BP and TPR in the initial period of orthostasis as it is shown in Table 5.

Moreover, the Valsalva index was smaller in patients of the group with progressive course of disease: 1.36 ± 0.11 and 1.82 ± 0.37 rel. un., *P* < 0.05 Therefore, sympathoadrenal hyperactivity, manifested as a significant increase in BP and TPR in response to the orthostasis against the decrease in efferent cardiac reactivity manifested in a decrease in the Valsalva index, is a predictor of the likely progression of hypertension.

### 3.4. Factors of New Case Development of Diabetes Mellitus

The analysis was carried out among patients who do not suffer from diabetes at the time of the first examination. There were four patients classified as having diabetes mellitus during the follow-up. The control group included 37 subjects considering that deceased patients and those who had diabetes before study were excluded from the analysis. It was found that all patients with newly developed diabetes regularly took statins, whereas only 15 patients (or 41%) took statins in the control group, *χ*^2^ = 5.13, *P* < 0.05. No hemodynamic parameters and indices of autonomic regulation were different between the groups.

## 4. Discussion

The course of our local one-center follow-up observation has shown that the prognosis for hypertensive patients depends on initial hemodynamic parameters and indices of autonomic regulation that are not taken to routine study in patients with cardiovascular diseases. We have found that in patients with the most severe prognosis, weaker efferent regulatory function on the heart and blood vessels and decreased neurogenic reactivity in response to physiological challenges against the background of the initial increase in peripheral vascular tone were observed. These data are consistent with the results of studies demonstrating a decrease in norepinephrine reuptake in myocardium [13] and an increase in TPR [10] in patients at high risk of sudden death. It is worth noting that the results in references [10, 13] were obtained in patients with significant heart failure, whereas in our study, neither drop in ejection fraction nor cardiomegaly was observed in any patient. Apparently, impairment of neurogenic control, which may occur in response to a decrease cardiac output, could be of independent significance for prognosis in patients with preserved left ventricular contractility.

In this study were performed easily reproducible and relatively simple tests that appear as clinical equivalent of more complex and expensive procedures. Thus, the Valsalva maneuver is a method for assessing the safety of the cardiac neurogenic regulation and can partly repeat such a complex method as myocardial scintigraphy with MIBG. Vasomotor function and sympathoadrenal reactivity reflect the cold vasoconstriction, the assessment of short-term BPV and tilt test. It is worth noting that most of the studies, which included the tilt test, were devoted to the study of orthostatic insufficiency, while an excessive increase in blood pressure in the upright position was ignored. Not enough attention was paid to assessment of the short-term BP during continuous BP recording. Nevertheless, an increase of this parameter in the respiratory range is associated with mortality risk, whereas its increase in the low-frequency range is a risk marker of the stroke.

The importance of higher BP variability in the respiratory range in patients with a negative prognosis of death from cardiovascular pathology remains insufficiently understood. However, most probable explanation of the revealed phenomenon is impairment of the autonomic nervous system function that provides diminishing of BP during breathing (the so-called cardiorespiratory conjugation). This impairment reflects heaviness of autonomic dysfunction.

Most likely, a negative prognosis regarding the development of stroke is associated with uncontrolled crisis type course of the arterial hypertension that is a consequence of two factors: increased vasomotor reactivity of BP and inadequate therapy of the disease. Evident markers of the negative prognosis are such simple indicators as orthostatic hypertension and increased variability of BP, the detection of which should alarm both the doctor and patient. Similar data describing the increase of short-term variability were also reported [14]. In this case, vasomotor reactivity may be one of the reasons for insufficient control of BP, thus requiring the use of individual approaches in the selection of therapy and disease control [15]. At the same time, strict control of blood pressure can improve the prognosis in patients with severe hypertension [16, 17] that is likely to be sought in this group of patients. Notably, the amount of antihypertensive drug groups prescribed for the treatment of stroke patients was the same as for others despite the higher BP and BPV in the former group.

It is worth pointing out that patients with negative prognosis characterized by reduced index Valsalva (which is a measure of cardiac reactivity), with orthostatic hypertension, and high BPV have high probability of hypertension progression. Therefore, they require increased attention from cardiologists to provide them with the best possible treatment. Probably, such a combination of indicators of autonomic control should alert the attending doctor to require careful control of risk factors. It is noteworthy that all patients received not only optimal antihypertensive, but also lipid-lowering therapy and amount and dose of drugs prescribed for the treatment of patients who did not significantly differ in groups with a more severe and normal prognosis. Moreover, there was no difference in the level of cholesterol between these groups.

Despite the fact that new cases of diabetes mellitus are associated with taking statins, which is consistent with the literature data [18], this condition is not accompanied by the development of severe complications of the hypertension over a sufficiently long period of observation. Considering that dyslipidemia contributes to the hypertension progression (which is especially pronounced under conditions of changes in cardiac and vascular reactivity), taking statins is justified for this group of patients with an unfavorable combination of risk factors in spite of the threat of the development of diabetes mellitus.

## 5. Perspectives

In this study, we have shown that along with well-known factors, such as hypercholesterolemia and high blood pressure level, the prognosis of hypertensive patients is influenced by regulatory and hemodynamic parameters. Variations of the indicators of autonomic regulation (such as diminished Valsalva indices and vasomotor reactivity, inadequate orthostatic reaction, increased BPV, and total peripheral resistance, especially in combination with high blood pressure and cholesterol level) are associated with the development of life-threatening complications in hypertensive patients as well. Although these indicators are very informative, they are not evaluated during the routine examination of patients with cardiovascular diseases. The cause of unfavorable changes of autonomic regulation of blood circulation in these patients remains unclear; nonetheless, their use as indicators of disease prognosis could be of great practical importance and serve as a signal for intensification of therapy. These changes require both the closer control of blood pressure and prescription of cholesterol-decreasing medicine. The main limitation of the study is that it was carried out in a small cohort of hypertensive patients. Therefore, further investigation of the observed phenomena should be performed in a larger population by multicenter study. Moreover, detailed research aimed to understand the reasons of unfavorable changes of systemic hemodynamic and autonomic regulation is to be carried out. It is also necessary to conduct a research, the purpose of which would be to study the long-term effects of adequate correction of hemodynamic parameters and lipid metabolism in terms of autonomic regulation and their relationship to outcomes in patients with hypertension not complicated by severe myocardial dysfunction.

## Figures and Tables

**Table 1:** Clinical characteristics of patients included in the study.

Associated clinical condition (ACC)	Occurrence (%) (number of cases)	Mean age ± SD (years)
All ACC	28 (51)	62.6 ± 4.2
Coronary heart disease	25 (45)	63.5 ± 4.2
Myocardial infarction	14 (25)	64.7 ± 5.7
Myocardial revascularization	13 (24)	60.8 ± 5.7
Stroke	2 (4)	63.0 ± 8/4
Severe atherosclerosis of brain arteries	2 (4)	57.5 ± 3.5
Chronic kidney disease stage IV	1 (2)	84
Without ACC	27 (49)	51.9 ± 9.9

**Table 2:** Questionnaire of the secondary examinations of patients.

	Question	Possible answer
1	Is patient alive?	Yes, no
*Have there been cardiovascular complications in the time that has elapsed since the examination?*		
2	Myocardial infarction	Yes, no
3	Stroke	Yes, no
4	Revascularization	Yes, no
5	New cases of diabetes mellitus	Yes, no
*Accepted therapy:*		
6	Regular use of antihypertensive drugs	Yes, no
7	The number of antihypertensive drugs, pcs	Value
8	Regular use of statins	Yes, no
*The therapy efficacy:*		
9	Subjective assessment of well-being	From 1 to 5
10	BP measurements at home: the mean of three last measures, mmHg	Value
11	The presence of hypertensive crises during the last three months	Yes, no
12	The level of total cholesterol in the last examination, mmol/l	Value

**Table 3:** Indices of systemic hemodynamics and autonomous regulation in patients with and without ACC during the initial examination.

Parameter	Without ACC	With ACC	*P*
Systolic BP (mmHg)	132 ± 16	134 ± 22	0.79
Diastolic BP (mmHg)	71 ± 11	65 ± 18	0.043
Heart rate (beat/min)	71 ± 11	66 ± 12	0.084
Valsalva index (rel. un.)	1.8 ± 0.4	1.51 ± 0.3	0.046
Hand grip (mmHg)	16.6 ± 6.7	14.1 ± 5.3	0.18
Cold vasoconstriction (rel. un.)	0.42 ± 0.15	0.29 ± 0.12	0.054
ABR (ms/mmHg)	7.8 ± 6.3	6.4 ± 4.0	0.31

**Table 4:** Outcomes of monitoring hypertensive patients with and without ACC in the course of the follow-up observation.

Outcome	Target organ damage (*n* = 28)	With associated clinical conditions (*n* = 28)	Significance of differences
Death	0	5	*P* < 0.05
Myocardial infarction	1	2	*P* > 0.05
Stroke	2	4	*P* > 0.05
Revascularization	1	5	*P* < 0.05
New cases of diabetes mellitus	1	3	*P* > 0.05
Progression of hypertension	4	—	

**Table 5:** Dynamics of arterial BP and TPR during 1 minute of the orthostasis in hypertensive patients without ACC at the time of initial examination.

	Progressive disease (*n* = 4)	Nonprogressive disease (*n* = 24)	*P*
Change of diastolic BP (mmHg)	6.64 ± 10.8	0.40 ± 6.3	0.05
Total peripheral resistance (rel. un.)	0.49 ± 0.80	0.05 ± 0.21	0.028

## Data Availability

The data sets supporting the conclusions of this article are included within the article.

## Abbreviations

ABR: Arterial baroreflex
ACC: Associated clinical conditions
BP: Blood pressure
BPV: Blood pressure variability
ECG: Electrocardiogram
TPR: Total peripheral resistance.
